# The Prevalence of Latent Tuberculosis Infection and Smear Positive Pulmonary Tuberculosis in People with Household Close Contact with Tuberculosis in North of Iran

**Published:** 2015-03

**Authors:** Mahmood Moosazadeh, Narges Khanjani, Mohammadreza Parsaee

**Affiliations:** 1 Health Sciences Research Center, Faculty of Health, Mazandaran University of Medical Sciences, Sari, Iran;; 2 Research Center for Environmental Health Engineering, Kerman Medical University, Kerman, Iran;; 3 Health Deputy, Kerman University of Medical Sciences, Kerman, Iran

**Keywords:** Tuberculosis, Household close contact, Iran, Prevalence

## Abstract

One of the recommended strategies for preventing tuberculosis is to screen high-risk populations with respect to Mycobacterium tuberculosis (TB) infection. The aim of the present study was to investigate latent infection and active tuberculosis in people with close household contact. It was a cross-sectional descriptive, analytical study with the sample size of 668 people from homes with one infected resident. In order to diagnose tuberculosis latent infection, the PPD test was done. To determine patients with smear-positive pulmonary tuberculosis, three sputum samples were taken from every patient and were examined using direct microscopy and culture. Data was analyzed by SPSS20 software. The prevalence of latent tuberculosis infection and smear-positive pulmonary tuberculosis were 42.8% and 0.9% respectively. The prevalence of latent tuberculosis infection and smear-positive pulmonary tuberculosis in people with close household contact were less than that of other studies. However, smear-positive pulmonary tuberculosis in people with close household contact was 199.5 times more than that of the general population.

## Introduction

Mycobacterium tuberculosis (TB) is mainly an airborne pathogen that is transferred from one person to another through respiration. About one third of the world’s population is estimated to be infected with Mycobacterium TB.^1^^-^^6^ Factors such as the source case, organism, environment and the person who has had contact with the source case determines the transfer of TB and the development of a new case.^1^^,^^2^^,^^7^^-^^9^

It has been proven that almost 30% of people with patient contact show some symptoms of this infection and half of them will develop the disease in the first two years.^10^ Previous studies have reported that latent infection and tuberculosis is more prevalent in home residents who have had contact with smear-positive pulmonary tuberculosis patients.^1^^,^^3^^,^^4^ In a study carried out in one of the rural areas of Pakistan, the prevalence of positive tuberculin skin test was 49.4% among people exposed to TB patients.^1^

One of the recommended strategies for preventing tuberculosis is to screen high-risk populations regarding Mycobacterium TB infection in order to identify people in need for chemoprophylaxis or treatment.^1^^,^^4^ Among those, people with close contact with patients are one of the high-risk groups regarding Mycobacterium TB infection. Hence, to offer suitable recommendations and guidelines to develop and improve the objectives of the tuberculosis control program, relatives of such patients have to be examined. The aim of this study was to determine the prevalence of latent tuberculosis infection and smear-positive pulmonary tuberculosis in people with household close contact with tuberculosis in the north of Iran.

## Materials and Methods

This cross-sectional study was carried out during 2010 and 2011 and the sampling was based on census. The populations under study were family members older than 6 years of age, having had contact with patients with smear-positive pulmonary tuberculosis in Mazandaran from 2006 to 2009. A “household close contact” was defined as someone who has lived for more than 30 days in the same house as an index case.^11^

The number of enrolled people participating in this study was 668. The data collection tool was based on a checklist that included variables such as age, sex, skin induration test, coughing more than two weeks, coughing with sputum, weight loss, and the result of sputum smear microscopic examination. 

After obtaining the agreement from the regional Health Deputy, an investigative team (including a physician, a disease expert, and a laboratory science expert) was formed and trained by our researchers to execute the investigation. Following the consent from the participants, demographic and field variables were inquired. Three sputum samples were taken from the individuals followed by direct sputum smear test at the Tuberculosis Laboratory of the City Health Department. The sputum culture was carried out at the Reference Laboratory of Mazandaran University of Medical Sciences. In order to ensure data accuracy of the sputum tests, 10% of negative sputum smear samples and 100% of positive sputum smear samples were re-examined at the Reference Laboratory. To evaluate smear-positive pulmonary tuberculosis, if one sputum smear sample and one sputum culture sample or at least two sputum smear samples of a person were positive, then that person was considered as a patient with smear-positive pulmonary tuberculosis.^5^^,^^6^ Moreover, all participants underwent a PPD test and their results were examined after 72 hours. If the induration diameter of the PPD test was 10 mm or more, this was considered as a latent tuberculosis infection.^1^ Data was entered into the SPSS20 software and the results were analyzed using descriptive statistics including mean, standard deviation, frequency, and analytical methods, including Chi-square or Fisher’s exact test (for comparison with fewer than 5 expected observations per cell) and *t *test. Crude and adjusted odds ratios were also calculated.

## Results

In total, 668 people being in close contact with smear-positive pulmonary tuberculosis patients were reviewed and examined; among which 44.5% (297 individuals) were men. The average age was 36.9±19.6. In men, the mean age was 33.9±18.8 and it was 39.3±19.9 in women. 1.5% of the participants had continuous cough for more than two weeks and 0.6% had weight loss. The prevalence of latent tuberculosis infection and smear-positive pulmonary tuberculosis was 42.8% and 0.9% respectively (table 1).

**Table 1:** Characteristics of people with close household contact with tuberculosis patients

**Characteristic**		**N**	**%**
Sex	Male	297	44.5
	Female	371	55.5
Age group	6-24	214	32
	25-44	225	33.7
	≥45	229	34.3
Cough more than two weeks	Yes	10	1.5
	No	658	98.5
Weight loss	Yes	4	0.6
	No	664	99.4
PPD test	<10 mm	382	57.2
	≥10 mm	286	42.8
Result of sputum smear	Yes	6	0.9
	No	662	99.1

Table 2 shows that among men and women, the prevalence of latent tuberculosis (41.8% vs. 43.7%) and smear-positive pulmonary tuberculosis (0.7% vs. 1.1%) had no significant difference (P=0.3 and P=0.4 respectively). The maximum prevalence of latent tuberculosis infection (47.7%) and smear-positive pulmonary tuberculosis (1.7%) was found in people aged 6-24 years and ≥45 years respectively, but these differences were not significant. People with continuous coughs for more than two weeks were meaningfully more than those without it in people with latent tuberculosis infection (90% vs. 42.1%) (P=0.003) and in patients with smear-positive pulmonary tuberculosis (30% vs. 0.5%) (P<0.001). Moreover, people with weight loss were more than people without weight loss in the latent infection group (100% vs. 42.5%) and was also higher in people with smear-positive pulmonary tuberculosis (75% vs. 0.5%) (P=0.03 and P<0.001 respectively).

**Table 2:** Characteristics of latent tuberculosis infection and smear positive tuberculosis patients in people with close household contact with tuberculosis patients (Chi-square test)

**Characteristic**		**PPD test with result of** **≥10** **N** **(%)**			**Positive sputum smear** **N** **(%)**		
		**Yes**	**No**	**P value**	**Yes**	**No**	**P value**
Sex	Male	124 (41.8)	173 (58.2)	0.3	(2) 0.7	295 (99.3)	0.4
	Female	162 (43.7)	209 (56.3)		(4) 1.1	367 (98.9)	
Age group	6-24	102 (47.7)	112 (52.3)	0.09	0	214 (0)	0. 1
	25-44	84 (37.3)	141 (62.7)		(2) 0.9	223 (99.1)	
	≥45	100 (43.7)	129 (56.3)		(4) 1.7	225 (98.3)	
Cough more than two weeks	Yes	9 (90)	1 (10)	0.003	(3) 30	7 (70)	<0.001
	No	277 (42.1)	381 (57.9)		(3) 0.5	655 (99.5)	
Weight loss	Yes	4 (100)	0	0.03	(3) 75	1 (25)	<0.001
	No	282 (42.5)	382 (57.5)		(3) 0.5	661 (99.5)	

## Discussion

Our findings show that the prevalence of both latent tuberculosis infection and smear-positive pulmonary tuberculosis among people with household close contacts was high in spite of the development of tuberculosis control program strategies.

Khalilzadeh et al., at Masih Daneshvari Pulmonary Diseases Research Center of Shahid Beheshti University (Iran),^12^ showed that the prevalence of latent tuberculosis infection and smear-positive pulmonary tuberculosis in people with close household contact was 38.2% and 4.8% respectively. Also, Khalilzadeh et al. in another study which was carried out during a two-year period (2002-2004) at the National Research Institute of Tuberculosis and Lung Disease of Iran, showed that among 224 close contacts, the initial Tuberculin Skin Test (TST) was positive (indurations>10 mm) in 16.5% of contacts and 7.6% had active positive smear.^11^

It is worth mentioning that the prevalence of smear-positive pulmonary tuberculosis among the general population of Mazandaran province was 4.5 in 100,000 people, 22.8 in 100,000 people in Golestan province (east of Mazandaran) and 7.7 in 100,000 people in Gilan Province (west of Mazandaran). Such incidence rates are less than the rate in people with household contacts.^5^^,^^13^

In one of the rural areas of Pakistan,^1^ the prevalence of positive tuberculin skin test (TST) was 49.4% among the people with close contact. In addition, the prevalence of Mycobacterium tuberculosis infection among the people with close household contacts in Spain and Peru was 44% and 55% respectively.^2^^,^^3^

In a systematic review that was carried out to determine the yield of household contact,^14^ the yield for all tuberculosis (bacteriologically confirmed and clinically diagnosed) was 4.5% (95% CI 4.3-4.8, I^ 2 ^=95.5%) of the investigated contacts and for cases with bacteriological confirmation the yield was 2.3% (95% CI 2.1-2.5, I^ 2 ^=96.6%). Latent tuberculosis infection was found in 51.4% (95% CI 50.6-52.2, I^ 2 ^=99.4%) of the investigated contacts.^14^

One reason for this lower prevalence can be related to the year where other studies are carried out which were around the previous six years or prior to 2008. More recently, governments have made improvements in procedures and strategies in the tuberculosis control program in order to achieve the millennium development objectives.^14^ Another probable reason is the heterogeneity of people who participated in these studies. It is proved in various studies^1^^,^^4^^,^^7^^,^^8^ that the contact duration, age of exposed people, relative’s sleeping site, severity of sputum smear examination of patients and BCG Scar status of contact people were variables that predict tuberculosis infection among contact people. These variables, however, were not accounted for in some studies, including the present study.

In this research, the prevalence of tuberculosis infection had no difference in men and women and matched the results of a study done by Rathi et al.^1^ Moreover, in another study,^3^ the prevalence of latent tuberculosis infection and smear-positive pulmonary tuberculosis increased as age increased; which was in line with our study.

One of the limitations in the current study was in examining people with household close contact, where we did not study the effect of the exposure time, contact duration, continuous exposure or sleeping in one place while inquiring infection. Another limitation was its cross-sectional nature where temporality (cause-and-effect relationship) could not be recognized. 

## Conclusion

In this research, the prevalence of smear-positive pulmonary tuberculosis in people with household close contact was 898 out of 100,000 people. This is 199.5 times more than the general population of this region in Iran. Therefore, it can be declared that investigating household close contacts is necessary and of great importance in the national tuberculosis control program. 

## References

[1] Rathi SK, Akhatar MH, Rahbar MH, Azam SI (2002). Prevalence and risk factors associated with tuberculin skin test positivity among household contacts of smear-positive pulmonary tuberculosis cases in Umerkot, Pakistan. Int J Tuberc Lung Dis.

[2] Madico G, Gilman RH, Checkley W, Cabrera L, Kohlstadt I, Kacena K (1995). Community infection ratio as an indicator for tuberculosis control. Lancet.

[3] Vidal R, Miravitlles M, Caylà JA, Torrella M, Martín N, de Gracia J (1997). A contagiousness study in 3071 familial contacts of tuberculosis patients. Med Clin Barc.

[4] Morrison J, Pai M, Hopewell PC (2008). Tuberculosis and latent tuberculosis infection in close contacts of people with pulmonary tuberculosis in low-income and middle-income countries: a systematic review and meta-analysis. Lancet Infect Dis.

[5] Nasehi MM, Moosazadeh M, Amiresmaeili MR, Parsaee MR, Nezammahalleh A (2012). The Epidemiology of Factors Associated with Screening and Treatment Outcomes of Patients with Smear Positive Pulmonary Tuberculosis: A Population-Based Study. J Mazandaran Univ Med Sci.

[6] Moosazadeh M, Jamshidi M, Amiresmaili MR, Nezammahalleh A (2012). A Comparison of Directly Observed Therapy and Self-Administered Therapy Strategies in Treatment of Pulmonary Tuberculosis: A Cohort Study in North of Iran. Middle-East Journal of Scientific Research.

[7] Lee MS, Leung CC, Kam KM, Wong MY, Leung MC, Tam CM (2008). Early and late tuberculosis risks among close contacts in Hong Kong. Int J Tuberc Lung Dis.

[8] Salinas C, Capelastegui A, Altube L, España PP, Díez R, Oribe M (2007). Longitudinal incidence of tuberculosis in a cohort of contacts: factors associated with the disease. Arch Bronconeumol.

[9] Ohno H, Ikegami Y, Kishida K, Yamamoto Y, Ikeda N, Taniguchi T (2008). A contact investigation of the transmission of Mycobacterium tuberculosis from a nurse working in a newborn nursery and maternity ward. J Infect Chemother.

[10] Tan MC, Marra CA, Sadatsafavi M, Marra F, Morán-Mendoza O, Moadebi S (2008). Cost-effectiveness of LTBI treatment for TB contacts in British Columbia. Value Health.

[11] Khalilzadeh S, Masjedi H, Hosseini M, Safavi A, Masjedi MR (2006). Transmission of Mycoba cterium tuberculosis to households of tuberculosis patients: a comprehensive contact tracing study. Arch Iranian Med.

[12] Khalilzadeh S, Zahirifard S, Masjedi H, Boloorsaz MR, Velayeti AA (2004). Prevalence of tuberculosis in close contacts of smear positive TB patients. Iranian journal of infectious diseases and tropical medicine.

[13] Moosazadeh M, Khanjani N (2012). The Existing Problems in the Tuberculosis Control Program of Iran: A Qualitative Study. Journal of Qualitative Research in Health Sciences.

[14] Fox GJ, Barry SE, Britton WJ, Marks GB (2013). Contact investigation for tuberculosis: a systematic review and meta-analysis. Eur Respir J.

